# Current Awareness on Comparative and Functional Genomics

**DOI:** 10.1002/cfg.228

**Published:** 2003-06


**Comparative and Functional Genomics**
*Comp Funct Genom* 2003; 4: 356-363
Published online in Wiley InterScience (www.interscience.wiley.com). **DOI** 10.1002/cfg.228

### Current awareness on comparative and functional genomics

### 1 Reviews & symposia

2002. Special issue on expression profiling within the central nervous system. *Neurochem Res* **27:** (10)
2002. Special issue: Comparative genomics. *Comp Biochem Physiol B Biochem Mol Biol* **133:** (4)
Awadalla P. 2003. The evolutionary genomics of pathogen recombination. *Nat Rev Genet* **4:** (1) 50.
Bacarese-Hamilton T, Bistoni F, Crisanti A. 2002. Protein microarrays: From serodiagnosis to whole proteome scale analysis of the immune response against pathogenic microorganisms. *Biotechniques* (Suppl) 24.
Bertucci F, Viens P, Hingamp P, Nasser V, Houlgatte R, Birnbaum D. 2003. Breast cancer revisited using DNA array-based gene expression profiling (Review). *Int J Cancer* **103:** (5) 565.
Butte A. 2002. The use and analysis of microarray data. *Nat Rev Drug Discov* **1:** (12) 951.
Cascio I, Rapaka RS. 2002. Structural biology and structural genomics/proteomics (Review). *J Pept Res* **60:** (6) 307.
Cheung VG, Spielman RS. 2002. The genetics of variation in gene expression. *Nat Genet* **32:** (Suppl) 522.
Chuaqui RF, Bonner RK, Best CJM, Gillespie JW, Flaig MJ, Hewitt SM, Phillips JL, Krizman DB, Tangrea MA, Ahram M *et al*. 2002. Post-analysis follow-up and validation of microarray experiments. *Nat Genet* **32:** (Suppl) 509.
Chung CH, Bernard PS, Perou CM. 2002. Molecular portraits and the family tree of cancer. *Nat Genet* **32:** (Suppl) 533.
Churchill GA. 2002. Fundamentals of experimental design for cDNA microarrays. *Nat Genet* **32:** (Suppl) 490.
Cullen P, Lorkowski S. 2002. DNA micro-arrays in medicine: Manufacturing techniques and potential applications. *Expert Opin Ther Patents* **12:** (12) 1783.
Del Vecchio VG, Wagner MA, Eschenbrenner M, Horn TA, Kraycer JA, Estock F, Elzer P, Mujer CV. 2002. *Brucella* proteomes: A review. *Vet Microbiol* **90:** (1-4 Suppl 1) 593.
Donadio S, Sosio M, Lancini G. 2002. Impact of the first *Streptomyces* genome sequence on the discovery and production of bioactive substances (Review). *Appl Microbiol Biotechnol* **60:** (4) 377.
Duyk GM. 2002. Sharper tools and simpler methods. *Nat Genet* **32:** (Suppl) 465.
Elia G, Silacci M, Scheurer S, Scheuermann J, Neri D. 2002. Affinity-capture reagents for protein arrays. *Trends Biotechnol* **20:** (12 Suppl) S19.
Fenyo D, Beavis RC. 2002. Informatics and data management in proteomics. *Trends Biotechnol* **20:** (12 Suppl) S35.
Flory MR, Griffin TJ, Martin D, Aebersold R. 2002. Advances in quantitative proteomics using stable isotope tags. *Trends Biotechnol* **20:** (12 Suppl) S23.
Fritz B, Raczniak GA. 2002. Bacterial genomics: Potential for antimicrobial drug discovery. *Biodrugs* **16:** (5) 331.
Gerhold DL, Jensen RV, Gullans SR. 2002. Better therapeutics through microarrays. *Nat Genet* **32:** (Suppl) 547.
Hallborn J, Carlsson R. 2002. Automated screening procedure for high-throughput generation of antibody fragments. *Biotechniques* (Suppl) 30.
Hancock WS, Wu SL, Stanley RR, Gombocz EA. 2002. Publishing large proteome datasets: Scientific policy meets emerging technologies. *Trends Biotechnol* **20:** (12 Suppl) S39.
Hirsch N, Zimmermann LB, Grainger RM. 2002. *Xenopus*, the next generation: *X. tropicalis* genetics and genomics (Review). *Dev Dyn* **225:** (4) 422.
Holloway AJ, Van Laar RK, Tothill RW, Bowtell DDL. 2002. Options available - from start to finish - for obtaining data from DNA microarrays. *Nat Genet* **32:** (Suppl) 481.
Hunter TC, Andon NL, Koller A, Yates JR, Haynes PA. 2002. The functional proteomics toolbox: Methods and applications. *J Chromatogr B* **782:** (1-2) 165.
James P. 2002. Chips for proteomics: A new tool or just hype? *Biotechniques* (Suppl) 4.
Klein SL, Strausberg RL, Wagner L, Pontius J, Clifton SW, Richardson P. 2002. Genetic and genomic tools for *Xenopus* research: The NIH *Xenopus* initiative (Review). *Dev Dyn* **225:** (4) 384.
Koshland DE, Hamadani K. 2002. Proteomics and models for enzyme cooperativity (Review). *J Biol Chem* **277:** (49) 46841.
Kusnezow W, Hoheisel JD. 2002. Antibody microarrays: Promises and problems. *Biotechniques* (Suppl) 14.
Lee YS, Mrksich M. 2002. Protein chips: From concept to practice. *Trends Biotechnol* **20:** (12 Suppl) S14.
Lin HN, Cornish VW. 2002. Screening and selection methods for large-scale analysis of protein function (Review). *Angew Chem Int Ed* **41:** (23) 4403.
Link AJ. 2002. Multidimensional peptide separations in proteomics. *Trends Biotechnol* **20:** (12 Suppl) S8.
Lubman DA, Kachman MT, Wang HX, Gong SY, Yan F, Hamler RL, O'Neil KA, Zhu K, Buchanan NS, Barder TJ. 2002. Two-dimensional liquid separations-mass mapping of proteins from human cancer cell lysates. *J Chromatogr B* **782:** (1-2) 183.
MacBeath G. 2002. Protein microarrays and proteomics. *Nat Genet* **32:** (Suppl) 526.
Maelicke A, Lubbert H. 2002. DEPD®, a high resolution gene expression profiling technique capable of identifying new drug targets in the central nervous system (Review). *J Recept Signal Transduct Res* **22:** (1-4) 283.
Maggio ET, Shenderovich M, Kagan R, Goddette D, Ramnarayan K. 2002. Structural pharmacogenomics, drug resistance and the design of anti-infective super-drugs. *Drug Discov Today* **7:** (24) 1214.
Masignani V, Rappuoli R, Pizza M. 2002. Reverse vaccinology: A genome-based approach for vaccine development. *Expert Opin Biol Ther* **2:** (8) 895.
Melese T, Hieter P. 2002. From genetics and genomics to drug discovery: Yeast rises to the challenge. *Trends Pharmacol Sci* **23:** (12) 544.
Michaux-Charachon S, Jumas-Bilak E, Allardet-Servent A, Bourg G, Boschiroli ML, Ramuz M, O'Callaghan D. 2002. The *Brucella* genome at the beginning of the post-genomic era. *Vet Microbiol* **90:** (1-4 Suppl 1) 581.
Nguyen DV, Arpat AB, Wang NY, Carroll RJ. 2002. DNA microarray experiments: Biological and technological aspects. *Biometrics* **58:** (4) 701.
O'Brien SJ, Menotti-Raymond M, Murphy WJ, Yuhki N. 2002. The Feline Genome Project. *Annu Rev Genet* **36:** 657.
Olson MV, Varki A. 2003. Sequencing the chimpanzee genome: Insights into human evolution and disease. *Nat Rev Genet* **4:** (1) 20.
Pardanani A, Wieben ED, Spelsberg TC, Tefferi A. 2002. Primer on medical genomics. Part IV: Expression proteomics. *Mayo Clin Proc* **77:** (11) 1185.
Petricoin EF, Hackett JL, Lesko LJ, Puri RK, Gutman SI, Chumakov K, Woodcock J, Feigal DW, Zoon KC, Sistare FD. 2002. Medical appli-

In order to keep subscribers up-to-date with the latest developments in their field, this current awareness service is provided by John Wiley & Sons and contains newly-published material on comparative and functional genomics. Each bibliography is divided into 16 sections. 1 Reviews & symposia; 2 General; 3 Large-scale sequencing and mapping; 4 Evolutionary genomics; 5 Comparative genomics; 6 Pathways, gene families and regulons; 7 Pharmacogenomics; 8 EST, cDNA and other clone resources; 9 Functional genomics; 10 Transcriptomics; 11 Proteomics; 12 Protein structural genomics; 13 Metabolomics; 14 Genomic approaches to development; 15 Technological advances; 16 Bioinformatics. Within each section, articles are listed in alphabetical order with respect to author. If, in the preceding period, no publications are located relevant to any one of these headings, that section will be omitted.

Copyright © 2003 John Wiley & Sons, Ltd.

cations of microarray technologies: A regulatory science perspective. *Nat Genet* **32:** (Suppl) 474.
Petricoin EF, Liotta LA. 2002. Proteomic analysis at the bedside: early detection of cancer. *Trends Biotechnol* **20:** (12 Suppl) S30.
Pollack JR, Iyer VR. 2002. Characterizing the physical genome. *Nat Genet* **32:** (Suppl) 515.
Posada D, Crandall KA, Holmes EC. 2002. Recombination in evolutionary genomics. *Annu Rev Genet* **36:** 75.
Quackenbush J. 2002. Microarray data normalization and transformation. *Nat Genet* **32:** (Suppl) 496.
Reinke V. 2002. Functional exploration of the *C. elegans* genome using DNA microarrays. *Nat Genet* **32:** (Suppl) 541.
Ricciardi-Castagnoli P, Granucci F. 2002. Interpretation of the complexity of innate immune responses by functional genomics. *Nat Rev Immunol* **2:** (11) 881.
Ryan TE, Patterson SD. 2002. Proteomics: Drug target discovery on an industrial scale. *Trends Biotechnol* **20:** (12 Suppl) S45.
Sanchez-Carbayo M. 2003. Use of high-throughput DNA microarrays to identify biomarkers for bladder cancer (Review). *Clin Chem* **49:** (1) 23.
Santori FR, Brown SM, Vukmanovic S. 2002. Genomics-based identification of self-ligands with T-cell receptor-specific biological activity. *Immunol Rev* **190:** (1) 146.
Scheetz T, Bartlett JA, Walters JD, Schutte BC, Casavant TL, McCray PB. 2002. Genomics-based approaches to gene discovery in innate immunity. *Immunol Rev* **190:** (1) 137.
Schlotterer C. 2003. Hitchhiking mapping: Functional genomics from the population genetics perspective. *Trends Genet* **19:** (1) 32.
Slonim DK. 2002. From patterns to pathways: Gene expression data analysis comes of age. *Nat Genet* **32:** (Suppl) 502.
Smith RD. 2002. Trends in mass spectrometry instrumentation for proteomics. *Trends Biotechnol* **20:** (12 Suppl) S3.
Sreekumar A, Chinnaiyan AM. 2002. Using protein microarrays to study cancer. *Biotechniques* (Suppl) 46.
Steinmetz LM, Deutschbauer AM. 2002. Gene function on a genomic scale. *J Chromatogr B* **782:** (1-2) 151.
Stoeckert CJ, Causton HC, Ball CA. 2002. Microarray databases: Standards and ontologies. *Nat Genet* **32:** (Suppl) 469.
Van der Weyden L, Adams DJ, Bradley A. 2002. Tools for targeted manipulation of the mouse genome (Review). *Physiol Genomics* **11:** (3) 133.
Waterhouse PM, Helliwell CA. 2003. Exploring plant genomes by RNA-induced gene silencing. *Nat Rev Genet* **4:** (1) 29.
Whittam TS, Bumbaugh AC. 2002. Inferences from whole-genome sequences of bacterial pathogens. *Curr Opin Genet Dev* **12:** (6) 719.
Wong IHN. 2003. Transcriptional profiling of circulating tumor cells: Quantification and cancer progression (Review). *Oncol Rep* **10:** (1) 229.

### 3 Large-scale sequencing and mapping

Del Vecchio VG, Kapatral V, Elzer P, Patra G, Mujer CV. 2002. The genome of *Brucella melitensis*. *Vet Microbiol* **90:** (1-4 Suppl 1) 587.
Heidelberg JF, Paulsen IT, Nelson KE, Gaidos EJ, Nelson WC, Read TD, Eisen JA, Seshadri R, Ward N, Methe B *et al*. 2002. Genome sequence of the dissimilatory metal ion-reducing bacterium *Shewanella oneidensis*. *Nat Biotechnol* **20:** (11) 1118.
Ohnishi M, Terajima J, Kurokawa K, Nakayama K, Murata T, Tamura K, Ogura Y, Watanabe H, Hayashi T. 2002. Genomic diversity of enterohemorrhagic *Escherichia coli* O157 revealed by whole genome PCR scanning. *Proc Natl Acad Sci U S A* **99:** (26) 17043.
Sasaki Y, Ishikawa J, Yamashita A, Oshima K, Kenri T, Furuya K, Yoshino C, Horino A, Shiba T, Sasaki T *et al*. 2002. The complete genomic sequence of *Mycoplasma penetrans*, an intracellular bacterial pathogen in humans. *Nucleic Acids Res* **30:** (23) 5293.
Stjepandic D, Weinel C, Hilbert H, Koo HL, Diehl F, Nelson KE, Tummler B, Hoheisel JD. 2002. The genome structure of *Pseudomonas putida*: High-resolution mapping and microarray analysis. *Environ Microbiol* **4:** (12) 819.
Waterston RH, Lindblad-Toh K, Birney E, Rogers J *et al*. 2002. Initial sequencing and comparative analysis of the mouse genome. *Nature* **420:** (6915) 520.
Weinel C, Nelson KE, Tummler B. 2002. Global features of the *Pseudomonas putida* KT2440 genome sequence. *Environ Microbiol* **4:** (12) 809.
Welch RA, Burland V, Plunkett G, Redford P, Roesch P, Rasko D, Buckles EL, Liou SR, Boutin A, Hackett J *et al*. 2002. Extensive mosaic structure revealed by the complete genome sequence of uropathogenic *Escherichia coli*. *Proc Natl Acad Sci U S A* **99:** (26) 17020.

### 4 Evolutionary genomics

Berg OG, Kurland CG. 2002. Evolution of microbial genomes: Sequence acquisition and loss. *Mol Biol Evol* **19:** (12) 2265.
Dehal P, Satou Y, Campbell RK, Chapman J, Degnan B, De Tomaso A, Davidson B, Di Gregorio A, Gelpke M, Goodstein DM *et al*. 2002. The draft genome of *Ciona intestinalis*: Insights into chordate and vertebrate origins. *Science* **298:** (5601) 2157.
Dunham MJ, Badrane H, Ferea T, Adams J, Brown PO, Rosenzweig F, Botstein D. 2002. Characteristic genome rearrangements in experimental evolution of *Saccharomyces cerevisiae*. *Proc Natl Acad Sci U S A* **99:** (25) 16144.
Fukushima A, Ikemura T, Kinouchi M, Oshima T, Kudo Y, Mori H, Kanaya S. 2002. Periodicity in prokaryotic and eukaryotic genomes identified by power spectrum analysis. *Gene* **300:** (1-2) 203.
Lecompte O, Ripp R, Thierry JC, Moras D, Poch O. 2002. Comparative analysis of ribosomal proteins in complete genomes: An example of reductive evolution at the domain scale. *Nucleic Acids Res* **30:** (24) 5382.
Ramakrishna W, Dubcovsky J, Park YJ, Busso C, Emberton J, San Miguel P, Bennetzen JL. 2002. Different types and rates of genome evolution detected by comparative sequence analysis of orthologous segments from four cereal genomes. *Genetics* **162:** (3) 1389.

### 5 Comparative genomics

Botina SG, Lysenko AM, Sukhodolets VV, Trenina MA. 2002. Comparison of genomes in *Streptococcus thermophilus* strains of different origin. *Microbiology-Engl Tr* **71:** (6) 707.
Chan K, Baker S, Kim CC, Detweiler CS, Dougan G, Falkow S. 2003. Genomic comparison of *Salmonella enterica* serovars and *Salmonella bongori* by use of an *S. enterica* serovar Typhimurium DNA microarray. *J Bacteriol* **185:** (2) 553.
Cockle PJ, Gordon SV, Lalvani A, Buddle BM, Hewinson RG, Vordermeier HM. 2002. Identification of novel *Mycobacterium tuberculosis* antigens with potential as diagnostic reagents or subunit vaccine candidates by comparative genomics. *Infect Immun* **70:** (12) 6996.
De Oliveira RC, Yanai GM, Muto NH, Leite DB, De Souza AA, Coletta HD, Machado MA, Nunes LR. 2002. Competitive hybridization on spotted microarrays as a tool to conduct comparative genomic analyses of *Xylella fastidiosa* strains. *FEMS Microbiol Lett* **216:** (1) 15.
Gitton Y, Dahmane N, Baik S, Altaba ARI, Neidhardt L, Scholze M, Herrmann BG, Kahlem P, Benkahla A, Schrinner S, Yildirimman R, Herwig R, Lehrach H, Yaspo ML. 2002. A gene expression map of human chromosome 21 orthologues in the mouse. *Nature* **420:** (6915) 586.
Hu Y, Hu N, Liu G. 2002. Complete genomes of two human hepatitis A virus isolates from China: Analysis and comparison with other isolates. *Acta Virol* **46:** (3) 153.
Kapfhammer D, Blass J, Evers S, Reidl J. 2002. *Vibrio cholerae* phage K139: Complete genome sequence and comparative genomics of related phages. *J Bacteriol* **184:** (23) 6592.
Nelson KE, Weinel C, Paulsen IT, Dodson RJ, Hilbert H, Dos Santos VAPM, Fouts DE, Gill SR, Pop M, Holmes M *et al*. 2002. Complete genome sequence and comparative analysis of the metabolically versatile *Pseudomonas putida* KT2440. *Environ Microbiol* **4:** (12) 799.
Perrin A, Bonacorsi S, Carbonnelle E, Talibi D, Dessen P, Nassif X, Tinsley C. 2002. Comparative genomics identifies the genetic islands that distinguish *Neisseria meningitidis*, the agent of cerebrospinal meningitis, from other *Neisseria* species. *Infect Immun* **70:** (12) 7063.
Reymond A, Marigo V, Yaylaoglu MB, Leoni A, Ucla C, Scamuffa N, Cacciopoli C, Dermitzakis ET, Lyle R, Banfi S, Eichele G, Antonarakis SE, Ballabio A. 2002. Human chromosome 21 gene expression atlas in the mouse. *Nature* **420:** (6915) 582.

Copyright © 2003 John Wiley & Sons, Ltd. 

### 6 Pathways, gene families and regulons

Gaballa A, Wang T, Ye RW, Helmann JD. 2002. Functional analysis of the *Bacillus subtilis* Zur regulon. *J Bacteriol* **184:** (23) 6508.

Rodionov DA, Vitreschak AG, Mironov AA, Gelfand MS. 2002. Comparative genomics of thiamin biosynthesis in procaryotes: New genes and regulatory mechanisms. *J Biol Chem* **277:** (50) 48949.

Zolla L, Rinalducci S, Timperio AM, Huber CG. 2002. Proteomics of light-harvesting proteins in different plant species. Analysis and comparison by liquid chromatography-electrospray ionization mass spectrometry. Photosystem I. *Plant Physiol* **130:** (4) 1938.

### 7 Pharmacogenomics

Brown RE, Kamal NR. 2002. The Reed-Sternberg cell: Molecular characterization by proteomic analysis with therapeutic implications. *Ann Clin Lab Sci* **32:** (4) 339.

Campanaro S, Romualdi C, Fanin M, Celegato B, Pacchioni B, Trevisan S, Laveder P, De Pitta C, Pegoraro E, Hayashi YK *et al*. 2002. Gene expression profiling in dysferlinopathies using a dedicated muscle microarray. *Hum Mol Genet* **11:** (26) 3283.

Campos AH, Zhao Y, Pollman MJ, Gibbons GH. 2003. DNA microarray profiling to identify angiotensin-responsive genes in vascular smooth muscle cells: Potential mediators of vascular disease. *Circ Res* **92:** (1) 111.

Cho YS, Kim MK, Cheadle C, Neary C, Park YG, Becker KG, Cho-Chung YS. 2002. A genomic-scale view of the cAMP response element-enhancer decoy: A tumor target-based genetic tool. *Proc Natl Acad Sci U S A* **99:** (24) 15626.

Chung EJ, Sung YK, Farooq M, Kim Y, Im S, Tak WY, Hwang YJ, Kim YI, Han HS, Kim JC *et al*. 2002. Gene expression profile analysis in human hepatocellular carcinoma by cDNA microarray. *Mol Cells* **14:** (3) 382.

Cline EI, Bicciato S, Di Bello C, Lingen MW. 2002. Prediction of *in vivo* synergistic activity of antiangiogenic compounds by gene expression profiling. *Cancer Res* **62:** (24) 7143.

Dyrskjot L, Thykjaer T, Kruhoffer M, Jensen JL, Marcussen N, Hamilton-Dutoit S, Wolf H, Orntoft TF. 2003. Identifying distinct classes of bladder carcinoma using microarrays. *Nat Genet* **33:** (1) 90.

Garcia JF, Camacho FI, Morente M, Fraga M, Montalban C, Alvaro T, Bellas C, Castano A, Diez A, Flores T *et al*. 2003. Hodgkin and Reed-Sternberg cells harbor alterations in the major tumor suppressor pathways and cell-cycle checkpoints: Analyses using tissue microarrays. *Blood* **101:** (2) 681.

Golembieski WA, Rempel SA. 2002. cDNA array analysis of SPARC-modulated changes in glioma gene expression. *J Neurooncol* **60:** (3) 213.

Guo X, Lui WO, Qian CN, Chen JD, Gray SG, Rhodes D, Haab B, Stanbridge E, Wang HM, Hong MH *et al*. 2002. Identifying cancer-related genes in nasopharyngeal carcinoma cell lines using DNA and mRNA expression profiling analyses. *Int J Oncol* **21:** (6) 1197.

Harding MA, Arden KC, Gildea JW, Gildea JJ, Perlman EJ, Viars C, Theodorescu D. 2002. Functional genomic comparison of lineage-related human bladder cancer cell lines with differing tumorigenic and metastatic potentials by spectral karyotyping, comparative genomic hybridization, and a novel method of positional expression profiling. *Cancer Res* **62:** (23) 6981.

Hasegawa S, Furukawa Y, Li M, Satoh S, Kato T, Watanabe T, Katagiri T, Tsunoda T, Yamaoka Y, Nakamura Y. 2002. Genome-wide analysis of gene expression in intestinal-type gastric cancers using a complementary DNA microarray representing 23,040 genes. *Cancer Res* **62:** (23) 7012.

Hoos A, Nissan A, Stojadinovic A, Shia J, Hedvat CV, Leung DHY, Paty PB, Klimstra D, Cordon-Cardo C, Wong WD. 2002. Tissue microarray molecular profiling of early, node-negative adenocarcinoma of the rectum; A comprehensive analysis. *Clin Cancer Res* **8:** (12) 3841.

Inoue H, Matsuyama A, Mimori K, Ueo H, Mori M. 2002. Prognostic score of gastric cancer determined by cDNA microarray. *Clin Cancer Res* **8:** (11) 3475.

Ishigaki S, Niwa J, Ando Y, Yoshihara T, Sawada K, Doyu M, Yamamoto M, Kato K, Yotsumoto Y, Sobue G. 2002. Differentially expressed genes in sporadic amyotrophic lateral sclerosis spinal cords: Screening by molecular indexing and subsequent cDNA microarray analysis. *FEBS Lett* **531:** (2) 354.

Juhasz O, Zhu Y, Garg R, Anisimov SV, Boheler KR. 2002. Analysis of altered genomic expression profiles in the senescent and diseased myocardium using cDNA microarrays. *Eur J Heart Fail* **4:** (6) 687.

Kim R, Trubetskoy A, Suzuki T, Jenkins NA, Copeland NG, Lenz J. 2003. Genome-based identification of cancer genes by proviral tagging in mouse retrovirus-induced T-cell lymphomas. *J Virol* **77:** (3) 2056.

Kokuryo T, Yamamoto T, Oda K, Kamiya J, Nimura Y, Senga T, Yasuda Y, Ohno Y, Nakanuma Y, Chen MF *et al*. 2003. Profiling of gene expression associated with hepatolithiasis by complementary DNA expression array. *Int J Oncol* **22:** (1) 175.

Kraus J, Pantel K, Pinkel D, Albertson DG, Speicher MR. 2003. High-resolution genomic profiling of occult micrometastic tumor cells. *Gene Chromosomes Cancer* **36:** (2) 159.

Ma J, Li J, Zhou J, Li XL, Tang K, Zhou M, Yang JB, Yan Q, Shen SR, Hu GX *et al*. 2002. Profiling genes differentially expressed in NGX6 overexpressed nasopharyngeal carcinoma cells by cDNA array. *J Cancer Res Clin Oncol* **128:** (12) 683.

Raghavan A, Ogilvie RL, Reilly C, Abelson ML, Raghavan S, Vasdewani J, Krathwohl M, Bohjanen PR. 2002. Genome-wide analysis of mRNA decay in resting and activated primary human T-lymphocytes. *Nucleic Acids Res* **30:** (24) 5529.

Robinson WH, Steinman L, Utz PJ. 2002. Protein and peptide array analysis of autoimmune disease. *Biotechniques* (Suppl) 66.

Sakakura C, Hagiwara A, Nakanishi M, Shimomura K, Takagi T, Yasuoka R, Fujita Y, Abe T, Ichikawa Y, Takahashi S *et al*. 2002. Differential gene expression profiles of gastric cancer cells established from primary tumour and malignant ascites. *Br J Cancer* **87:** (10) 1153.

Sanchez-Carbayo M, Socci ND, Charytonowicz E, Lu ML, Prystowsky M, Childs G, Cordon-Cardo C. 2002. Molecular profiling of bladder cancer using cDNA microarrays: Defining histogenesis and biological phenotypes. *Cancer Res* **62:** (23) 6973.

Schuppe-Koistinen I, Frisk AL, Janzon L. 2002. Molecular profiling of hepatotoxicity induced by a aminoguanidine carboxylate in the rat: Gene expression profiling. *Toxicology* **179:** (3) 197.

Simon R, Radmacher MD, Dobbin K, McShane LM. 2003. Pitfalls in the use of DNA microarray data for diagnostic and prognostic classification (Commentary). *J Nat Cancer Inst* **95:** (1) 14.

Uchida T, Fukawa A, Uchida M, Fujita K, Saito K. 2002. Application of a novel protein biochip technology for detection and identification of rheumatoid arthritis biomarkers in synovial fluid. *J Proteome Res* **1:** (6) 495.

Verwanger T, Sanovic R, Aberger F, Frischauf AM, Krammer B. 2002. Gene expression pattern following photodynamic treatment of the carcinoma cell line A-431 analysed by cDNA arrays. *Int J Oncol* **21:** (6) 1353.

### 8 EST, cDNA and other clone resources

Faccioli P, Lagonigro MS, De Cecco L, Stanca AM, Alberici R, Terzi V. 2002. Analysis of differential expression of barley ESTs during cold acclimatization using microarray technology. *Plant Biol* **4:** (5) 630.

Felipe MSS, Andrade RV, Petrofeza SS, Maranhao AQ, Torres FAG *et al*. 2003. Transcriptome characterization of the dimorphic and pathogenic fungus *Paracoccidioides brasiliensis* by EST analysis. *Yeast* **20:** (3) 263.

Fischer K, Holt DC, Harumal P, Currie BJ, Walton SF, Kemp DJ. 2003. Generation and characterization of cDNA clones from *Sarcoptes scabiei* var. *hominis* for an expressed sequence tag library: Identification of homologues of house dust mite allergens. *Am J Trop Med Hyg* **68:** (1) 61.

Journet EP, Van Tuinen D, Gouzy J, Crespeau H, Carreau V, Farmer MJ, Niebel A, Schiex T, Jaillon O, Chatagnier O *et al*. 2002. Exploring root symbiotic programs in the model legume *Medicago truncatula* using EST analysis. *Nucleic Acids Res* **30:** (24) 5579.

Junqueirade-Azevedo IDM, Ho PL. 2002. A survey of gene expression and diversity in the venom glands of the pitviper snake *Bothrops insularis* through the generation of expressed sequence tags (ESTs). *Gene* **299:** (1-2) 279.

Kan ZY, States D, Gish W. 2002. Selecting for functional alternative splices in ESTs. *Genome Res* **12:** (12) 1837.

Copyright © 2003 John Wiley & Sons, Ltd. 

Michiels F, Van Es H, Van Rompaey L, Merchiers P, Francken B, Pittois K, Van der Schueren J, Brys R, Vandersmissen J, Beirinckx F *et al*. 2002. Arrayed adenoviral expression libraries for functional screening. *Nat Biotechnol* **20:** (11) 1154.

Milla MAR, Butler E, Huete AR, Wilson CF, Anderson O, Gustafson JP. 2002. Expressed sequence tag-based gene expression analysis under aluminum stress in rye. *Plant Physiol* **130:** (4) 1706.

Ng ST, Jangi MS, Shirley MW, Tomley FM, Wan KL. 2002. Comparative EST analyses provide insights into gene expression in two asexual developmental stages of *Eimeria tenella*. *Exp Parasitol* **101:** (2-3) 168.

Okazaki Y, Furuno M, Kasukawa T, Adachi J, Bono H, Kondo S, Nikaido I, Osato N, Saito R, Suzuki H *et al*. 2002. Analysis of the mouse transcriptome based on functional annotation of 60,770 full-length cDNAs. *Nature* **420:** (6915) 563.

Wirtz MK, Samples JR, Xu H, Severson T, Acott TS. 2002. Expression profile and genome location of cDNA clones from an infant human trabecular meshwork cell library. *Invest Ophthalmol Vis Sci* **43:** (12) 3698.

Yao JB, Coussens PM, Saama P, Suchyta S, Ernst CW. 2002. Generation of expressed sequence tags from a normalized porcine skeletal muscle cDNA library. *Anim Biotechnol* **13:** (2) 211.

### 9 Functional genomics

Ashrafi K, Chang FY, Watts JL, Fraser AG, Kamath RS, Ahringer J, Ruvkun G. 2003. Genome-wide RNAi analysis of *Caenorhabditis elegans* fat regulatory genes. *Nature* **421:** (6920) 268.

Belli SI, Witcombe D, Wallach MG, Smith NC. 2002. Functional genomics of *gam56*: Characterisation of the role of a 56 kilodalton sexual stage antigen in oocyst wall formation in *Eimeria maxima*. *Int J Parasitol* **32:** (14) 1727.

Genter MB, Burman DM, Vijayakumar S, Ebert CL, Aronow BJ. 2002. Genomic analysis of alachlor-induced oncogenesis in rat olfactory mucosa. *Physiol Genomics* **12:** (1) 35.

Hofmann HA. 2003. Functional genomics of neural and behavioral plasticity. *J Neurobiol* **54:** (1) 272.

Kamath RS, Fraser AG, Dong Y, Poulin G, Durbin R, Gotta M, Kanapin A, Le Bot N, Moreno S, Sohrmann M *et al*. 2003. Systematic functional analysis of the *Caenorhabditis elegans* genome using RNAi. *Nature* **421:** (6920) 231.

Li KC. 2002. Genome-wide coexpression dynamics: Theory and application. *Proc Natl Acad Sci U S A* **99:** (26) 16875.

Pekarik V, Bourikas D, Miglino N, Joset P, Preiswerk S, Stoeckli ET. 2003. Screening for gene function in chicken embryo using RNAi and electroporation. *Nat Biotechnol* **21:** (1) 93.

Peters DG, Zhang XC, Benos PV, Heidrich-O'Hare E, Ferrell RE. 2002. Genomic analysis of immediate/early response to shear stress in human coronary artery endothelial cells. *Physiol Genomics* **12:** (1) 25.

Walhout AJM, Reboul J, Shtanko O, Bertin N, Vaglio P, Ge H, Lee H, Doucette-Stamm L, Gunsalus KC, Schetter AJ *et al*. 2002. Integrating interactome, phenome, and transcriptome mapping data for the *C. elegans* germline. *Curr Biol* **12:** (22) 1952.

Wuchty S. 2002. Interaction and domain networks of yeast. *Proteomics* **2:** (12) 1715.

Zhang J, Schneider C, Ottmers L, Rodriguez R, Day A, Markwardt J, Schneider BL. 2002. Genomic scale mutant hunt identifies cell size homeostasis genes in *S. cerevisiae*. *Curr Biol* **12:** (23) 1992.

### I0 Transcriptomics

Boyce JD, Wilkie I, Harper M, Paustian ML, Kapur V, Adler B. 2002. Genomic scale analysis of *Pasteurella multocida* gene expression during growth within the natural chicken host. *Infect Immun* **70:** (12) 6871.

Cavallaro S, D'Agata V, Manickam P, Dufour F, Alkon DL. 2002. Memory-specific temporal profiles of gene expression in the hippocampus. *Proc Natl Acad Sci U S A* **99:** (25) 16279.

Ceriani MF, Hogenesch JB, Yanovsky M, Panda S, Straume M, Kay SA. 2002. Genome-wide expression analysis in *Drosophila* reveals genes controlling circadian behavior. *J Neurosci* **22:** (21) 9305.

Chen TB, Bjourson AJ, Orr DF, Kwok H, Rao PF, Ivanyi C, Shaw C. 2002. Unmasking venom gland transcriptomes in reptile venoms. *Anal Biochem* **311:** (2) 152.

Dietrich G, Kurz S, Hubner C, Aepinus C, Theiss S, Guckenberger M, Panzner U, Weber J, Frosch M. 2003. Transcriptome analysis of *Neisseria meningitidis* during infection. *J Bacteriol* **185:** (1) 155.

Dong H, Toyoda N, Yoneyama H, Kurachi M, Kasahara T, Kobayashi Y, Inadera H, Hashimoto S, Matsushima K. 2002. Gene expression profile analysis of the mouse liver during bacteria-induced fulminant hepatitis by a cDNA microarray system. *Biochem Biophys Res Commun* **298:** (5) 675.

Ebrahimi B, Dutia BM, Roberts KL, Garcia-Ramirez JJ, Dickinson P, Stewart JP, Ghazal P, Roy DJ, Nash AA. 2003. Transcriptome profile of murine gammaherpesvirus-68 lytic infection. *J Gen Virol* **84:** (1) 99.

Eckardt NE. 2002. *Chlamydomonas* chloroplast genome and transcriptome analysis. *Plant Cell* **14:** (11) 2657.

FitzPatrick DR, Ramsay J, McGill NI, Shade M, Carothers AD, Hastie ND. 2002. Transcriptome analysis of human autosomal trisomy. *Hum Mol Genet* **11:** (26) 3249.

Goda H, Shimada Y, Asami T, Fujioka S, Yoshida S. 2002. Microarray analysis of brassinosteroid-regulated genes in *Arabidopsis*. *Plant Physiol* **130:** (3) 1319.

Hoth S, Morgante M, Sanchez JP, Hanafey MK, Tingey SV, Chua NH. 2002. Genome-wide gene expression profiling in *Arabidopsis thaliana* reveals new targets of abscisic acid and largely impaired gene regulation in the *abi1-1* mutant. *J Cell Sci* **115:** (24) 4891.

Jones JO, Arvin AM. 2003. Microarray analysis of host cell gene transcription in response to varicella-zoster virus infection of human T-cells and fibroblasts *in vitro* and SCIDhu skin xenografts *in vivo*. *J Virol* **77:** (2) 1268.

Kaan T, Homuth G, Mader U, Bandow J, Schweder T. 2002. Genome-wide transcriptional profiling of the *Bacillus subtilis* cold-shock response. *Microbiology* **148:** (11) 3441.

Kreps JA, Wu TJ, Chang HS, Zhu T, Wang X, Harper JF. 2002. Transcriptome changes for *Arabidopsis* in response to salt, osmotic, and cold stress. *Plant Physiol* **130:** (4) 2129.

Kumar-Sinha C, Ignatoski KW, Lippman ME, Ethier SP, Chinnaiyan AM. 2003. Transcriptome analysis of HER2 reveals a molecular connection to fatty acid synthesis. *Cancer Res* **63:** (1) 132.

Li CH, Zhu YQ, Meng YL, Wang JW, Xu KX, Zhang TZ, Chen XY. 2002. Isolation of genes preferentially expressed in cotton fibers by cDNA filter arrays and RT-PCR. *Plant Sci* **163:** (6) 1113.

Liang XB, Zhang H, Zhou AY, Wang HY. 2003. Screening for functional genes related to a novel gene, AngRem104, in human mesangial cells by cDNA microarray. *Biotechnol Lett* **25:** (2) 139.

McLean DJ, Friel PJ, Pouchnik D, Griswold MD. 2002. Oligonucleotide microarray analysis of gene expression in follicle-stimulating hormone-treated rat Sertoli cells. *Mol Endocrinol* **16:** (12) 2780.

Nakajima T, Inagaki N, Tanaka H, Tanaka A, Yoshikawa M, Tamari M, Hasegawa K, Matsumoto K, Tachimoto H, Ebisawa M *et al*. 2002. Marked increase in CC chemokine gene expression in both human and mouse mast cell transcriptomes following Fc ε receptor I cross-linking: An interspecies comparison. *Blood* **100:** (12) 3861.

Olesen K, Felding T, Gjermansen C, Hansen J. 2002. The dynamics of the *Saccharomyces carlsbergensis* brewing yeast transcriptome during a production-scale lager beer fermentation. *FEMS Yeast Res* **2:** (4) 563.

Park WY, Hwang CI, Im CN, Kang MJ, Woo JH, Kim JH, Kim YS, Kim JH, Kim H, Kim KA *et al*. 2002. Identification of radiation-specific responses from gene expression profile. *Oncogene* **21:** (55) 8521.

Paustian ML, May BJ, Cao DW, Boley D, Kapur V. 2002. Transcriptional response of *Pasteurella multocida* to defined iron sources. *J Bacteriol* **184:** (23) 6714.

Rossel JB, Wilson IW, Pogson BJ. 2002. Global changes in gene expression in response to high light in *Arabidopsis*. *Plant Physiol* **130:** (3) 1109.

Sahara T, Goda T, Ohgiya S. 2002. Comprehensive expression analysis of time-dependent genetic responses in yeast cells to low temperature. *J Biol Chem* **277:** (51) 50015.

Saiura A, Sugawara Y, Harihara Y, Sata M, Hamakubo T, Kodama T, Makuuchi M. 2002. Gene expression profile during acute rejection in rat-to-mouse concordant cardiac xenograft by means of DNA microarray. *Transpl Int* **15:** (11) 535.

Schlaak JF, Hilkens CMU, Costa-Pereira AP, Strobl B, Aberger F, Frischauf AM, Kerr IM. 2002. Cell-type and donor-specific transcriptional responses to interferon-α: Use of customized gene arrays. *J Biol Chem* **277:** (51) 49428.

Copyright © 2003 John Wiley & Sons, Ltd. 

Schoondermark-Stolk SA, Ter Schure EG, Verrips CT, Verkleij AJ, Boonstra J. 2002. Identification of salt-induced genes of *Zygosaccharomyces rouxii* by using *Saccharomyces cerevisiae* GeneFilters®. *FEMS Yeast Res* **2:** (4) 525.

Singh U, Shah P. 2002. Investigating amoebic pathogenesis using *Entamoeba histolytica* DNA microarrays. *J Biosci* **27:** (6 Suppl 3) 595.

Steenman M, Chen YW, Le Cunff M, Lamirault G, Varro A, Hoffman E, Leger JJ. 2003. Transcriptomal analysis of failing and nonfailing human hearts. *Physiol Genomics* **12:** (2) 97.

Van Ijperen C, Kuhnert P, Frey J, Clewley JP. 2002. Virulence typing of *Escherichia coli* using microarrays. *Mol Cell Probes* **16:** (5) 371.

Vaudry D, Chen Y, Ravni A, Hamelink C, Elkahloun AG, Eiden LE. 2002. Analysis of the PC12 cell transcriptome after differentiation with pituitary adenylate cyclase-activating polypeptide (PACAP). *J Neurochem* **83:** (6) 1272.

Wang HY, Ma LG, Habashi J, Li JM, Zhao HY, Deng XW. 2002. Analysis of far-red light-regulated genome expression profiles of phytochrome A pathway mutants in *Arabidopsis*. *Plant J* **32:** (5) 723.

Wasserman SM, Mehraban F, Komuves LG, Yang RB, Tomlinson JE, Zhang Y, Spriggs F, Topper JN. 2002. Gene expression profile of human endothelial cells exposed to sustained fluid shear stress. *Physiol Genomics* **12:** (1) 13.

Zhang L, Zhang Y, Zhou YM, Zhao YJ, Zhou YX, Cheng J. 2002. Expression profiling of the response of *Saccharomyces cerevisiae* to 5-fluorocytosine using a DNA microarray. *Int J Antimicrob Agents* **20:** (6) 444.

Zhao XS, Gallardo TD, Lin L, Schageman JJ, Shohet RV. 2002. Transcriptional mapping and genomic analysis of the cardiac atria and ventricles. *Physiol Genomics* **12:** (1) 53.

Zhao YH, Chen BPC, Miao H, Yuan SL, Li YS, Hu YL, Rocke DM, Chien S. 2002. Improved significance test for DNA microarray data: Temporal effects of shear stress on endothelial genes. *Physiol Genomics* **12:** (1) 1.

### II Proteomics

Aksu S, Scheler C, Focks N, Leenders F, Theuring F, Salnikow J, Jungblut PR. 2002. An iterative calibration method with prediction of post-translational modifications for the construction of a two-dimensional electrophoresis database of mouse mammary gland proteins. *Proteomics* **2:** (10) 1452.

Bajo M, Fruehauf J, Kim SH, Fountoulakis M, Lubec G. 2002. Proteomic evaluation of intermediary metabolism enzyme proteins in fetal Down's syndrome cerebral cortex. *Proteomics* **2:** (11) 1539.

Balmer Y, Koller A, Del Val G, Manieri W, Schurmann P, Buchanan BB. 2003. Proteomics gives insight into the regulatory function of chloroplast thioredoxins. *Proc Natl Acad Sci U S A* **100:** (1) 370.

Bernert G, Fountoulakis M, Lubec G. 2002. Manifold decreased protein levels of matrin 3, reduced motor protein HMP and hlark in fetal Down's syndrome brain. *Proteomics* **2:** (12) 1752.

Chelius D, Huhmer AFR, Shieh CH, Lehmberg E, Traina JA, Slattery TK, Pungor E. 2002. Analysis of the adenovirus type 5 proteome by liquid chromatography and tandem mass spectrometry methods. *J Proteome Res* **1:** (6) 501.

Clements LD, Streips UN, Miller BS. 2002. Differential proteomic analysis of *Bacillus subtilis* nitrate respiration and fermentation in defined medium. *Proteomics* **2:** (12) 1724.

Doyle SA, Murphy MB, Massi JM, Richardson PM. 2002. High-throughput proteomics: A flexible and efficient pipeline for protein production. *J Proteome Res* **1:** (6) 531.

Forler D, Kocher T, Rode M, Gentzel M, Izaurralde E, Wilm M. 2003. An efficient protein complex purification method for functional proteomics in higher eukaryotes. *Nat Biotechnol* **21:** (1) 89.

Fountoulakis M, Juranville JF, Dierssen M, Lubec G. 2002. Proteomic analysis of the fetal brain. *Proteomics* **2:** (11) 1547.

Fountoulakis M, Suter L. 2002. Proteomic analysis of the rat liver. *J Chromatogr B* **782:** (1-2) 197.

Gajendran N, Frey JR, Lefkovits I, Kuhn L, Fountoulakis M, Krapfenbauer K, Brenner HR. 2002. Proteomic analysis of secreted muscle components: Search for factors involved in neuromuscular synapse formation. *Proteomics* **2:** (11) 1601.

Gianazza E, Eberini I, Arnoldi A, Wait R, Sirtori CR. 2003. A proteomic investigation of isolated soy proteins with variable effects in experimental and clinical studies. *J Nutr* **133:** (1) 9.

Giometti CS, Reich C, Tollaksen S, Babnigg G, Lim HJ, Zhu WH, Yates J, Olsen G. 2002. Global analysis of a "simple" proteome: *Methanococcus jannaschii*. *J Chromatogr B* **782:** (1-2) 227.

Heim S, Lleo MD, Bonato B, Guzman CA, Canepari P. 2002. The viable but nonculturable state and starvation are different stress responses of *Enterococcus faecalis*, as determined by proteome analysis. *J Bacteriol* **184:** (23) 6739.

Heine G, Zucht HD, Schuhmann MU, Burger K, Jurgens M, Zumkeller M, Schneekloth CG, Hampel H, Schulz-Knappe P, Selle H. 2002. High-resolution peptide mapping of cerebrospinal fluid: A novel concept for diagnosis and research in central nervous system diseases. *J Chromatogr B* **782:** (1-2) 353.

Hesketh AR, Chandra G, Shaw AD, Rowland JJ, Kell DB, Bibb MJ, Chater KF. 2002. Primary and secondary metabolism, and post-translational protein modifications, as portrayed by proteomic analysis of *Streptomyces coelicolor*. *Mol Microbiol* **46:** (4) 917.

Huang HM, Tam MF, Tam TCS, Chen DH, Hsieh ML, Li C. 2002. Proteomic analysis of stable protein methylation in lymphoblastoid cells. *J Biochem* **132:** (5) 813.

Kellner R, Lichtenfels R, Atkins D, Bukur J, Ackermann A, Beck J, Brenner W, Melchior S, Seliger B. 2002. Targeting of tumor associated antigens in renal cell carcinoma using proteome-based analysis and their clinical significance. *Proteomics* **2:** (12) 1743.

Kim J, Kim SH, Lee SU, Ha GH, Kang DG, Ha NY, Ahn JS, Cho HY, Kang SJ, Lee YJ, Hong SC, Ha WS, Bae JM, Lee CW, Kim JW. 2002. Proteome analysis of human liver tumor tissue by two-dimensional gel electrophoresis and matrix assisted laser desorption/ionization-mass spectrometry for identification of disease-related proteins. *Electrophoresis* **23:** (24) 4142.

Kuwana R, Kasahara Y, Fujibayashi M, Takamatsu H, Ogasawara N, Watabe K. 2002. Proteomics characterization of novel spore proteins of *Bacillus subtilis*. *Microbiology* **148:** (12) 3971.

Le Bouder-Langevin S, Capron-Montaland I, De Rosa R, Labedan B. 2002. A strategy to retrieve the whole set of protein modules in microbial proteomes. *Genome Res* **12:** (12) 1961.

Levi-Schaffer F, Temkin V, Simon HU, Kettman JR, Frey JR, Lefkovits I. 2002. Proteomic analysis of human eosinophil activation mediated by mast cells, granulocyte macrophage colony stimulating factor and tumor necrosis factor $\alpha$. *Proteomics* **2:** (11) 1616.

Lopez JL, Marina A, Alvarez G, Vazquez J. 2002. Application of proteomics for fast identification of species-specific peptides from marine species. *Proteomics* **2:** (12) 1658.

Magnusson LU, Nystrom T, Farewell A. 2003. Underproduction of $\sigma^{70}$ mimics a stringent response: A proteome approach. *J Biol Chem* **278:** (2) 968.

Man WJ, White IR, Bryant D, Bugelski P, Camilleri P, Cutler P, Heald G, Lord PG, Wood J, Kramer K. 2002. Protein expression analysis of drug-mediated hepatoxicity in the Sprague-Dawley rat. *Proteomics* **2:** (11) 1577.

Michaud GA, Snyder M. 2002. Proteomic approaches for the global analysis of proteins. *Biotechniques* **33:** (6) 1308.

Mitterdorfer G, Mayer HK, Kneifel W, Viernstein H. 2002. Protein fingerprinting of *Saccharomyces* isolates with therapeutic relevance using one- and two-dimensional electrophoresis. *Proteomics* **2:** (11) 1532.

Navarre C, Degand H, Bennett KL, Crawford JS, Mortz E, Boutry M. 2002. Subproteomics: Identification of plasma membrane proteins from the yeast *Saccharomyces cerevisiae*. *Proteomics* **2:** (12) 1706.

Orr DF, Chen TB, Johnsen AH, Chalk R, Buchanan KD, Sloan JM, Rao PF, Shaw C. 2002. The spectrum of endogenous human chromogranin A-derived peptides identified using a modified proteomic strategy. *Proteomics* **2:** (11) 1586.

Radzikowski L, Nesic L, Hansen HB, Jacobsen S, Sondergaard I. 2002. Comparison of ethanol-soluble proteins from different rye (*Secale cereale*) varieties by two-dimensional electrophoresis. *Electrophoresis* **23:** (24) 4157.

Sarto C, Valsecchi C, Mocarelli P. 2002. Renal cell carcinoma: Handling and treatment. *Proteomics* **2:** (11) 1627.

Scherl A, Coute Y, Deon C, Calle A, Kindbeiter K, Sanchez JC, Greco A, Hochstrasse D, Diaz JJ. 2002. Functional proteomic analysis of human nucleolus. *Mol Biol Cell* **13:** (11) 4100.

Seliger B, Kellner R. 2002. Design of proteome-based studies in combination with serology for the identification of biomarkers and novel targets. *Proteomics* **2:** (12) 1641.

Copyright © 2003 John Wiley & Sons, Ltd. 

Simon WJ, Hall JJ, Suzuki I, Murata N, Slabas AR. 2002. Proteomic study of the soluble proteins from the unicellular cyanobacterium *Synechocystis* sp PCC6803 using automated matrix-assisted laser desorption/ionization-time of flight peptide mass fingerprinting. *Proteomics* **2:** (12) 1735.

Sinha S, Arora S, Kosalai K, Namane A, Pym AS, Cole ST. 2002. Proteome analysis of the plasma membrane of *Mycobacterium tuberculosis*. *Comp Funct Genom* **3:** (6) 470.

Sparre T, Christensen UB, Larsen PM, Fey SJ, Wrzesinski K, Roepstorff P, Mandrup-Poulsen T, Pociot F, Karlsen AE, Nerup J. 2002. IL-1β induced protein changes in diabetes prone BB rat islets of Langerhans identified by proteome analysis. *Diabetologia* **45:** (11) 1550.

Thiellement H, Zivy M, Plomion C. 2002. Combining proteomic and genetic studies in plants. *J Chromatogr B* **782:** (1-2) 137.

Thoren K, Gustafsson E, Clevnert A, Larsson T, Bergstrom J, Nilsson CL. 2002. Proteomic study of non-typable *Haemophilus influenzae*. *J Chromatogr B* **782:** (1-2) 219.

Van den Heuvel L, Farhoud MH, Wevers RA, Van Engelen B, Smeitink JAM. 2003. Proteomics and neuromuscular diseases: Theoretical concept and first results. *Ann Clin Biochem* **40:** (1) 9.

Wilkins MR. 2002. What do we want from proteomics in the detection and avoidance of adverse drug reactions. *Toxicol Lett* **127:** (1-3) 245.

Wulfkuhle JD, Sgroi DC, Krutzsch H, McLean K, McGarvey K, Knowlton M, Chen S, Shu HJ, Sahin A, Kurek R *et al*. 2002. Proteomics of human breast ductal carcinoma *in situ*. *Cancer Res* **62:** (22) 6740.

Xiong L, Regnier FE. 2002. Use of a lectin affinity selector in the search for unusual glycosylation in proteomics. *J Chromatogr B* **782:** (1-2) 405.

Yan JX, Devenish AT, Wait R, Stone T, Lewis S, Fowler S. 2002. Fluorescence two-dimensional difference gel electrophoresis and mass spectrometry based proteomic analysis of *Escherichia coli*. *Proteomics* **2:** (12) 1682.

Yoon HJ, Feoktistova A, Wolfe BA, Jennings JL, Link AJ, Gould KL. 2002. Proteomics analysis identifies new components of the fission and budding yeast anaphase-promoting complexes. *Curr Biol* **12:** (23) 2048.

Yu CJ, Lin YF, Chiang BL, Chow LP. 2003. Proteomics and immunological analysis of a novel shrimp allergen, Pen m 2. *J Immunol* **170:** (1) 445.

Zheng PP, Kros JM, Sillevis-Smitt PAE, Luider TM. 2003. Proteomics in primary brain tumors. *Front Biosci* **8:** (Jan) D451.

Zhu Y, Wu ZH, Tang ZM, Lu ZH. 2002. HeLa cell adhesion on various collagen-grafted surfaces. *J Proteome Res* **1:** (6) 559.

Zuo X, Hembach P, Echan L, Speicher DW. 2002. Enhanced analysis of human breast cancer proteomes using micro-scale solution isoelectrofocusing combined with high resolution 1D and 2D gels. *J Chromatogr B* **782:** (1-2) 253.

### I2 Protein structural genomics

Ding HT, Ren H, Chen Q, Fang G, Li LF, Li R, Wang R, Jia XY, Liang YH, Hu MH *et al*. 2002. Parallel cloning, expression, purification and Crystallograllization of human proteins for structural genomics. *Acta Crystallogr D Biol Crystallogr* **58:** (12) 2102.

Eswaramoorthy S, Gerchman S, Graziano V, Kycia H, Studier FW, Swaminathan S. 2003. Structure of a yeast hypothetical protein selected by a structural genomics approach. *Acta Crystallogr D-Biol Crystallogr* **59:** (1) 127.

Jacchieri SG. 2002. Foldability and the amino acid compositions of exons and introns. *J Proteome Res* **1:** (6) 515.

Kuznetsov VY, Ivanov YD, Bykov VA, Saunin SA, Fedorov IA, Lemeshko SV, Hoa HB, Archakov AI. 2002. Atomic force microscopy detection of molecular complexes in multiprotein P450cam containing monooxygenase system. *Proteomics* **2:** (12) 1699.

### I3 Metabolomics

Jimenez JI, Minambres B, Garcia JL, Diaz E. 2002. Genomic analysis of the aromatic catabolic pathways from *Pseudomonas putida* KT2440. *Environ Microbiol* **4:** (12) 824.

Papin JA, Price ND, Palsson BO. 2002. Extreme pathway lengths and reaction participation in genome-scale metabolic networks. *Genome Res* **12:** (12) 1889.

Rodriguez-Concepcion M, Boronat A. 2002. Elucidation of the methylerythritol phosphate pathway for isoprenoid biosynthesis in bacteria and plastids. A metabolic milestone achieved through genomics. *Plant Physiol* **130:** (3) 1079.

### I4 Genomic approaches to development

Dalbies-Tran R, Mermillod P. 2003. Use of heterologous complementary DNA array screening to analyze bovine oocyte transcriptome and its evolution during *in vitro* maturation. *Biol Reprod* **68:** (1) 252.

Demura T, Tashiro G, Horiguchi G, Kishimoto N, Kubo M, Matsuoka N, Minami A, Nagata-Hiwatashi M, Nakamura K, Okamura Y *et al*. 2002. Visualization by comprehensive microarray analysis of gene expression programs during transdifferentiation of mesophyll cells into xylem cells. *Proc Natl Acad Sci U S A* **99:** (24) 15794.

Gammill LS, Bronner-Fraser M. 2002. Genomic analysis of neural crest induction. *Development* **129:** (24) 5731.

Shen X, Collier JM, Hlaing M, Zhang L, Delshad EH, Bristow J, Bernstein HS. 2003. Genome-wide examination of myoblast cell cycle withdrawal during differentiation. *Dev Dyn* **226:** (1) 128.

Tanaka TS, Kunath T, Kimber WL, Jaradat SA, Stagg CA, Usuda M, Yokota T, Niwa H, Rossant J, Ko MSH. 2002. Gene expression profiling of embryo-derived stem cell reveals candidate genes associated with pluripotency and lineage specificity. *Genome Res* **12:** (12) 1921.

### I5 Technological advances

Carboni L, Piubelli C, Righetti PG, Jansson B, Domenici E. 2002. Proteomic analysis of rat brain tissue: Comparison of protocols for two-dimensional gel electrophoresis analysis based on different solubilizing agents. *Electrophoresis* **23:** (24) 4132.

Chrisman PA, McLuckey SA. 2002. Dissociations of disulfide-linked gaseous polypeptide/protein anions: Ion chemistry with implications for protein identification and characterization. *J Proteome Res* **1:** (6) 549.

Consolandi C, Castiglioni B, Bordoni R, Busti E, Battaglia C, Bernardi LR, De Bellis G. 2002. Two efficient polymeric chemical platforms for oligonucleotide microarray preparation. *Nucleosides Nucleotides* **21:** (8-9) 561.

Craft D, Doucette A, Li L. 2002. Microcolumn capture and digestion of proteins combined with mass spectrometry for protein identification. *J Proteome Res* **1:** (6) 537.

Creasy DM, Cottrell JS. 2002. Error tolerant searching of uninterpreted tandem mass spectrometry data. *Proteomics* **2:** (10) 1426.

Detter JC, Jett JM, Lucas SM, Dalin E, Arellano AR, Wang M, Nelson JR, Chapman J, Lou YI, Rokhsar D *et al*. 2002. Isothermal strand-displacement amplification for high-throughput genomics. *Genomics* **80:** (6) 691.

Fang Y, Frutos AG, Webb B, Hong YL, Ferrie A, Lai F, Lahiri J. 2002. Membrane biochips. *Biotechniques* (Suppl) 62.

Fetsch PA, Simone NL, Bryant-Greenwood PK, Marincola FM, Filie AC, Petricoin EF, Liotta LA, Abati A. 2002. Proteomic evaluation of archival cytologic material using SELDI affinity mass spectrometry: Potential for diagnostic applications. *Am J Clin Pathol* **118:** (6) 870.

Gay S, Binz PA, Hochstrasser DF, Appel RD. 2002. Peptide mass fingerprinting peak intensity prediction: Extracting knowledge from spectra. *Proteomics* **2:** (10) 1374.

Giddings MC, Shah AA, Gesteland R, Moore B. 2003. Genome-based peptide fingerprint scanning. *Proc Natl Acad Sci U S A* **100:** (1) 20.

Gorg A, Boguth G, Kopf A, Reil G, Parlar H, Weiss W. 2002. Sample prefractionation with Sephadex isoelectric focusing prior to narrow pH range two-dimensional gels. *Proteomics* **2:** (12) 1652.

He QY, Yip TT, Li MY, Chiu JF. 2003. Proteomic analyses of arsenic-induced cell transformation with SELDI-TOF ProteinChip®. *J Cell Biochem* **88:** (1) 1.

He Y, Zhong WW, Yeung ES. 2002. Multiplexed on-column protein digestion and capillary electrophoresis for high-throughput comprehensive peptide mapping. *J Chromatogr B* **782:** (1-2) 331.

Henningsen R, Gale BL, Straub KM, De Nagel DC. 2002. Application of zwitterionic detergents to the solubilization of integral membrane proteins for two-dimensional gel electrophoresis and mass spectrome-

Copyright © 2003 John Wiley & Sons, Ltd. 

try. *Proteomics* **2:** (11) 1479.
Hesse J, Wechselberger C, Sonnleitner M, Schindler H, Schutz GJ. 2002. Single-molecule reader for proteomics and genomics. *J Chromatogr B* **782:** (1-2) 127.
Hollemeyer K, Heinzle E, Tholey A. 2002. Identification of oxidized methionine residues in peptides containing two methionine residues by derivatization and matrix-assisted laser desorption/ionization mass spectrometry. *Proteomics* **2:** (11) 1524.
Howard EI, Cachau RE. 2002. Ink-jet printer heads for ultra-small-drop protein crystallography. *Biotechniques* **33:** (6) 1302.
Ishihama Y, Rappsilber J, Andersen JS, Mann M. 2002. Microcolumns with self-assembled particle frits for proteomics. *J Chromatogr A* **979:** (1-2) 233.
Johnson L, Gershon PD. 2002. Direct detection of protein thiol derivatization by PAGE. *Biotechniques* **33:** (6) 1292.
Karty JA, Ireland MME, Brun YV, Reilly JP. 2002. Artifacts and unassigned masses encountered in peptide mass mapping. *J Chromatogr B* **782:** (1-2) 363.
Keller BO, Wang ZP, Li L. 2002. Low-mass proteome analysis based on liquid chromatography fractionation, nanoliter protein concentration/digestion, and microspot matrix-assisted laser desorption/ionization mass spectrometry. *J Chromatogr B* **782:** (1-2) 317.
Lee MLT, Whitmore GA. 2002. Power and sample size for DNA microarray studies. *Stat Med* **21:** (23) 3543.
Lee YJ, Hoaglund-Hyzera CS, Barnes CAS, Hilderbrand AE, Valentine SJ, Clemmer DE. 2002. Development of high-throughput liquid chromatography injected ion mobility quadrupole time-of-flight techniques for analysis of complex peptide mixtures. *J Chromatogr B* **782:** (1-2) 343.
Liu HJ, Berger SJ, Chakraborty AB, Plumb RS, Cohen SA. 2002. Multidimensional chromatography coupled to electrospray ionization time-of-flight mass spectrometry as an alternative to two-dimensional gels for the identification and analysis of complex mixtures of intact proteins. *J Chromatogr B* **782:** (1-2) 267.
Miller LD, Long PM, Wong L, Mukherjee S, McShane LM, Liu ET. 2002. Optimal gene expression analysis by microarrays. *Cancer Cell* **2:** (5) 353.
Mineki R, Taka H, Fujimura T, Kikkawa M, Shindo N, Murayama K. 2002. *In situ* alkylation with acrylamide for identification of cysteinyl residues in proteins during one-and two-dimensional sodium dodecyl sulphate-polyacrylamide gel electrophoresis. *Proteomics* **2:** (12) 1672.
Miyaji T, Hewitt SM, Liotta LA, Star RA. 2002. Frozen protein arrays: A new method for arraying and detecting recombinant and native tissue proteins. *Proteomics* **2:** (11) 1489.
Muller M, Gras R, Binz PA, Hochstrasser DF, Appel RD. 2002. Molecular scanner experiment with human plasma: Improving protein identification by using intensity distributions of matching peptide masses. *Proteomics* **2:** (10) 1413.
Olsson I, Larsson K, Palmgren R, Bjellqvist B. 2002. Organic disulfides as a means to generate streak-free two-dimensional maps with narrow range basic immobilized pH gradient strips as first product dimension. *Proteomics* **2:** (11) 1630.
Patel K, Stein R, Benvenuti S, Zvelebil MJ. 2002. Combinatorial use of mRNA and two-dimensional electrophoresis expression data to choose relevant features for mass spectrometric identification. *Proteomics* **2:** (10) 1464.
Peterson DS, Rohr T, Svec F, Frechet JMJ. 2002. High-throughput peptide mass mapping using a microdevice containing trypsin immobilized on a porous polymer monolith coupled to MALDI TOF and ESI TOF mass spectrometers. *J Proteome Res* **1:** (6) 563.
Ruotolo BT, Gillig KJ, Stone EG, Russell DH. 2002. Peak capacity of ion mobility mass spectrometry: Separation of peptides in helium buffer gas. *J Chromatogr B* **782:** (1-2) 385.
Salmi J, Aittokallio T, Westerholm J, Griese M, Rosengren A, Nyman A, Lahesmaa R, Nevalainen O. 2002. Hierarchical grid transformation for image warping in the analysis of two-dimensional electrophoresis gels. *Proteomics* **2:** (11) 1504.
Schwenk JM, Stoll D, Templin MF, Joos TO. 2002. Cell microarrays: An emerging technology for the characterization of antibodies. *Biotechniques* (Suppl) 54.
Simon R, Sauter G. 2002. Tissue microarrays for miniaturized high-throughput molecular profiling of tumors. *Exp Hematol* **30:** (12) 1365.
Steinhauer C, Wingren C, Hager ACM, Borrebaeck CAK. 2002. Single framework recombinant antibody fragments designed for protein chip applications. *Biotechniques* (Suppl) 38.
Vilen H, Aalto JM, Kassinen A, Paulin L, Savilahti H. 2003. A direct transposon insertion tool for modification and functional analysis of viral genomes. *J Virol* **77:** (1) 123.
Walcher W, Oberacher H, Troiani S, Holzl G, Oefner P, Zolla L, Huber CG. 2002. Monolithic capillary columns for liquid chromatography-electrospray ionization mass spectrometry in proteomic and genomic research. *J Chromatogr B* **782:** (1-2) 111.
Wang YK, Quinn DF, Ma ZX, Fu EW. 2002. Inverse labeling-mass spectrometry for the rapid identification of differentially expressed protein markers/targets. *J Chromatogr B* **782:** (1-2) 291.
Waugh R, Dear PH, Powell W, Machray GC. 2002. Physical education: New technologies for mapping plant genomes. *Trends Plant Sci* **7:** (12) 521.
Weinberger SR, Boschetti E, Santambien P, Brenac V. 2002. Surface-enhanced laser desorption-ionization retentate chromatography™ mass spectrometry (SELDI-RC-MS): A new method for rapid development of process chromatography conditions. *J Chromatogr B* **782:** (1-2) 307.
Willats WGT, Rasmussen SE, Kristensen T, Mikkelsen JD, Knox JP. 2002. Sugar-coated microarrays: A novel slide surface for the high-throughput analysis of glycans. *Proteomics* **2:** (12) 1666.
Wilson NL, Schulz BL, Karlsson NG, Packer NH. 2002. Sequential analysis of *N*- and *O*-linked glycosylation of 2D-PAGE separated glycoproteins. *J Proteome Res* **1:** (6) 521.
Wind M, Kelm O, Nigg EA, Lehmann WD. 2002. Identification of phosphorylation sites in the polo-like kinases Plx1 and Plk1 by a novel strategy based on element and electrospray high resolution mass spectrometry. *Proteomics* **2:** (11) 1516.
Wirth U, Muller D, Schindler P, Lange J, Van Ostrum J. 2002. Post-translational modification detection using metastable ions in reflector matrix-assisted laser desorption/ionization-time of flight mass spectrometry. *Proteomics* **2:** (10) 1445.
Wool A, Smilansky Z. 2002. Precalibration of matrix-assisted laser desorption/ionization-time of flight spectra for peptide mass fingerprinting. *Proteomics* **2:** (10) 1365.

### I6 Bioinformatics

Aires-de Sousa J, Aires-de Sousa L. 2003. Representation of DNA sequences with virtual potentials and their processing by (SEQREP) Kohonen self-organizing maps. *Bioinformatics* **19:** (1) 30.
Anbazhagan R. 2003. Microarray data assembler. *Bioinformatics* **19:** (1) 157.
Bhan A, Galas DJ, Dewey TG. 2002. A duplication growth model of gene expression networks. *Bioinformatics* **18:** (11) 1486.
Bijlani R, Cheng YH, Pearce DA, Brooks AI, Ogihara M. 2003. Prediction of biologically significant components from microarray data: Independently Consistent Expression Discriminator (ICED). *Bioinformatics* **19:** (1) 62.
Black MA, Doerge RW. 2002. Calculation of the minimum number of replicate spots required for detection of significant gene expression fold change in microarray experiments. *Bioinformatics* **18:** (12) 1609.
Bock JR, Gough DA. 2003. Whole-proteome interaction mining. *Bioinformatics* **19:** (1) 125.
Carpi A, Di Maira G, Vedovato M, Rossi V, Naccari T, Floriduz M, Terzi M, Filippini F. 2002. Comparative proteome bioinformatics: Identification of a whole complement of putative protein tyrosine kinases in the model flowering plant *Arabidopsis thaliana*. *Proteomics* **2:** (11) 1494.
Chen X, Li M, Ma B, Tromp J. 2002. DNACompress: Fast and effective DNA sequence compression. *Bioinformatics* **18:** (12) 1696.
Colantuoni C, Henry G, Zeger S, Pevsner J. 2002. SNOMAD (Standardization and NOrmalization of MicroArray Data): Web-accessible gene expression data analysis. *Bioinformatics* **18:** (11) 1540.
Creighton C, Hanash S. 2003. Mining gene expression databases for association rules. *Bioinformatics* **19:** (1) 79.
Culhane AC, Perriere G, Considine EC, Cotter TG, Higgins DG. 2002. Between-group analysis of microarray data. *Bioinformatics* **18:** (12) 1600.
Cummings L, Riley L, Black L, Souvorov A, Resenchuk S, Dondoshansky I, Tatusova T. 2002. Genomic BLAST: Custom-defined virtual databases for complete and unfinished genomes. *FEMS Microbiol*

Copyright © 2003 John Wiley & Sons, Ltd. 

*Lett* **216:** (2) 133.
De Hoon MJL, Imoto S, Miyano S. 2002. Statistical analysis of a small set of time-ordered gene expression data using linear splines. *Bioinformatics* **18:** (11) 1477.
De Oliveira T, Miller R, Tarin M, Cassol S. 2003. An integrated genetic data environment (GDE)-based LINUX interface for analysis of HIV-1 and other microbial sequences. *Bioinformatics* **19:** (1) 153.
Deutsch JM. 2003. Evolutionary algorithms for finding optimal gene sets in microarray prediction. *Bioinformatics* **19:** (1) 45.
Dobbin K, Simon R. 2002. Comparison of microarray designs for class comparison and class discovery. *Bioinformatics* **18:** (11) 1438.
Dufresne G, Takacs L, Heus HC, Codani JJ, Duval M. 2002. Patent searches for genetic sequences: How to retrieve relevant records from patented sequence databases. *Nat Biotechnol* **20:** (12) 1269.
Ehlers A, Osborne J, Slack S, Roper RL, Upton C. 2002. Poxvirus orthologous clusters (POCs). *Bioinformatics* **18:** (11) 1544.
Elnitski L, Riemer C, Petrykowska H, Florea L, Schwartz S, Miller W, Hardison B. 2002. PipTools: A computational toolkit to annotate and analyze pairwise comparisons of genomic sequences. *Genomics* **80:** (6) 681.
Everitt R, Minnema SE, Wride MA, Koster CS, Hance JE, Mansergh FC, Rancourt DE. 2002. RED: The analysis, management and dissemination of expressed sequence tags. *Bioinformatics* **18:** (12) 1692.
Gaizauskas R, Demetriou G, Artymiuk PJ, Willett P. 2003. Protein structures and information extraction from biological tests: The PASTA system. *Bioinformatics* **19:** (1) 135.
Gattiker A, Bienvenut WV, Bairoch A, Gasteiger E. 2002. FindPept, a tool to identify unmatched masses in peptide mass fingerprinting protein identification. *Proteomics* **2:** (10) 1435.
Gilks WR, Audit B, De Angelis D, Tsoka S, Ouzounis CA. 2002. Modeling the percolation of annotation errors in a database of protein sequences. *Bioinformatics* **18:** (12) 1641.
Grishin VN, Grishin NV. 2002. Euclidian space and grouping of biological objects. *Bioinformatics* **18:** (11) 1523.
Howe K, Bateman A, Durbin R. 2002. QuickTree: Building huge Neighbour-Joining trees of protein sequences. *Bioinformatics* **18:** (11) 1546.
Hubbell E, Liu WM, Mei R. 2002. Robust estimators for expression analysis. *Bioinformatics* **18:** (12) 1585.
Ilyin VA, Pieper U, Stuart AC, Marti-Renom MA, McMahan L, Sali A. 2003. ModView, visualization of multiple protein sequences and structures. *Bioinformatics* **19:** (1) 165.
Keith JM, Adams P, Bryant D, Kroese DP, Mitchelson KR, Cochran DAE, Lala GH. 2002. A simulated annealing algorithm for finding consensus sequences. *Bioinformatics* **18:** (11) 1494.
Kirschner M, Pujol G, Radu A. 2002. Oligonucleotide microarray data mining: Search for age-dependent gene expression. *Biochem Biophys Res Commun* **298:** (5) 772.
Kulkarni AV, Williams NS, Lian Y, Wren JD, Mittelman D, Pertsemlidis A, Garner HR. 2002. ARROGANT: An application to manipulate large gene collections. *Bioinformatics* **18:** (11) 1410.
Lee KE, Sha NJ, Dougherty ER, Vannucci M, Mallick BK. 2003. Gene selection: A Bayesian variable selection approach. *Bioinformatics* **19:** (1) 90.
Lee W, Chen SL. 2002. Genome-tools: A flexible package for genome sequence analysis. *Biotechniques* **33:** (6) 1334.
Leluk J, Konieczny L, Roterman I. 2003. Search for structural similarity in proteins. *Bioinformatics* **19:** (1) 117.
Lemon WJ, Palatini JJT, Krahe R, Wright FA. 2002. Theoretical and experimental comparisons of gene expression indexes for oligonucleotide arrays. *Bioinformatics* **18:** (11) 1470.
Lester PJ, Hubbard SJ. 2002. Comparative bioinformatic analysis of complete proteomes and protein parameters for cross-species identification in proteomics. *Proteomics* **2:** (10) 1392.
Li JY, Liu HQ, Downing JR, Yeoh AEJ, Wong LS. 2003. Simple rules underlying gene expression profiles of more than six subtypes of acute lymphoblastic leukemia (ALL) patients. *Bioinformatics* **19:** (1) 71.
Lin YL, Huang XQ, Jiang T, Chao KM. 2003. MAVG: Locating non-overlapping maximum average segments in a given sequence. *Bioinformatics* **19:** (1) 151.
Liu WM, Mei R, Di X, Ryder TB, Hubbell E, Dee S, Webster TA, Harrington CA, Ho MH, Baid J *et al*. 2002. Analysis of high density expression microarrays with signed-rank call algorithms. *Bioinformatics* **18:** (12) 1593.
Martoglio AM, Miskin JW, Smith SK, MacKay DJC. 2002. A decomposition model to track gene expression signatures: Preview on observer-independent classification of ovarian cancer. *Bioinformatics* **18:** (12) 1617.
Mavroudi S, Papadimitriou S, Bezerianos A. 2002. Gene expression data analysis with a dynamically extended self-organized map that exploits class information. *Bioinformatics* **18:** (11) 1446.
McIndoe RA, Lanzen A, Hurtz K. 2003. MADGE: Scalable distributed data management software for cDNA microarrays. *Bioinformatics* **19:** (1) 87.
McKay SJ, Jones SJM. 2002. AcePrimer: automation of PCR primer design based on gene structure. *Bioinformatics* **18:** (11) 1538.
McShane LM, Radmacher MD, Freidlin B, Yu R, Li MC, Simon R. 2002. Methods for assessing reproducibility of clustering patterns observed in analyses of microarray data. *Bioinformatics* **18:** (11) 1462.
Mrowka R, Schuchhardt J, Gille C. 2002. Oligodb: Interactive design of oligo DNA for transcription profiling of human genes. *Bioinformatics* **18:** (12) 1686.
Nguyen DV, Rocke DM. 2002. Partial least squares proportional hazard regression for application to DNA microarray survival data. *Bioinformatics* **18:** (12) 1625.
Ogurtsov AY, Roytberg MA, Shabalina SA, Kondrashov AS. 2002. OWEN: Aligning long collinear regions of genomes. *Bioinformatics* **18:** (12) 1703.
Ooi CH, Tan P. 2003. Genetic algorithms applied to multi-class prediction for the analysis of gene expression data. *Bioinformatics* **19:** (1) 37.
Otu HH, Sayood K. 2003. A divide-and-conquer approach to fragment assembly. *Bioinformatics* **19:** (1) 22.
Park PJ, Butte AJ, Kohane IS. 2002. Comparing expression profiles of genes with similar promoter regions. *Bioinformatics* **18:** (12) 1575.
Reese JT, Pearson WR. 2002. Empirical determination of effective gap penalties for sequence comparison. *Bioinformatics* **18:** (11) 1500.
Romualdi C, Bortoluzzi S, D'Alessi F, Danieli GA. 2003. IDEG6: A web tool for detection of differentially expressed genes in multiple tag sampling experiments. *Physiol Genomics* **12:** (2) 159.
Roytberg MA, Ogurtsov AY, Shabalina SA, Kondrashov AS. 2002. A hierarchical approach to aligning collinear regions of genomes. *Bioinformatics* **18:** (12) 1673.
Safran M, Solomon I, Shmueli O, Lapidot M, Shen-Orr S, Adato A, Ben Dor U, Esterman N, Rosen N, Peter I *et al*. 2002. GeneCards™ 2002: towards a complete, object-oriented, human gene compendium. *Bioinformatics* **18:** (11) 1542.
Sasik R, Calvo E, Corbeil J. 2002. Statistical analysis of high-density oligonucleotide arrays: A multiplicative noise model. *Bioinformatics* **18:** (12) 1633.
Sheik SS, Sundararajan P, Hussain ASZ, Sekar K. 2002. Ramachandran plot on the web. *Bioinformatics* **18:** (11) 1548.
Shepherd AJ, Martin NJ, Johnson RG, Kellam P, Orengo CA. 2002. PFDB: A generic protein family database integrating the CATH domain structure database with sequence based protein family resources. *Bioinformatics* **18:** (12) 1666.
Stojanovic N, Chang JL, Lehoczky J, Zody MC, Dewar K. 2002. Identification of mixups among DNA sequencing plates. *Bioinformatics* **18:** (11) 1418.
Troyanskaya OG, Garber ME, Brown PO, Botstein D, Altman RB. 2002. Nonparametric methods for identifying differentially expressed genes in microarray data. *Bioinformatics* **18:** (11) 1454.
Wernisch L, Kendall SL, Soneji S, Wietzorrek A, Parish T, Hinds J, Butcher PD, Stoker NG. 2003. Analysis of whole-genome microarray replicates using mixed models. *Bioinformatics* **19:** (1) 53.
Xu D, Li GS, Wu LY, Zhou JZ, Xu Y. 2002. PRIMEGENS: Robust and efficient design of gene-specific probes for microarray analysis. *Bioinformatics* **18:** (11) 1432.
Yang AS, Wang LY. 2002. Local structure-based sequence profile database for local and global protein structure predictions. *Bioinformatics* **18:** (12) 1650.
Yang AS. 2002. Structure-dependent sequence alignment for remotely related proteins. *Bioinformatics* **18:** (12) 1658.
Zhang JH, Wu LY, Zhang XS. 2003. Reconstruction of DNA sequencing by hybridization. *Bioinformatics* **19:** (1) 14.
Zhang JH, Carey V, Gentleman R. 2003. An extensible application for assembling annotation for genomic data. *Bioinformatics* **19:** (1) 155.
Zhang N, Aebersold R, Schwilkowski B. 2002. ProbID: A probabilistic algorithm to identify peptides through sequence database searching using tandem mass spectral data. *Proteomics* **2:** (10) 1406.

Copyright © 2003 John Wiley & Sons, Ltd. 
